# Nutrient Utilization and Requirements in Sheep and Goats Raised Under Different Systems and Fed Low Nutritional Novel Feeds for Meat Production

**DOI:** 10.3390/ani15182658

**Published:** 2025-09-11

**Authors:** Osman Mahgoub, Nur El Huda I. E. Osman, Christopher D. Lu

**Affiliations:** 1 Animal Science Consulting, London, ON L9T 7W2, Canada; hudaisam@gmail.com; 2 College of Agriculture and Natural Resources, University of Hawaii, Hilo, HI 96720, USA; chrislu@hawaii.edu

**Keywords:** sheep, goats, nutrient utilization, nutrient requirements, novel feeds, low quality feeds

## Abstract

Sheep and goats are essential in many parts of the world, providing meat, milk, and fiber while also aiding rural and low-income communities. The cost of feeding is a major factor in the overall expenses of animal production. Therefore, it is crucial to improve nutrient utilization and accurately assess nutrient needs. Sheep and goats in different parts of the world are raised under different systems influenced by variations in environmental conditions. In tropical areas, sheep and goats often consume low-quality diets that do not meet their production requirements. These substandard diets, which include high-fiber feeds like straw or hay, are commonly provided to small ruminants and can adversely impact their feed intake, digestion, and nutrient absorption. To address these shortcomings, these feeds are usually supplemented with high-energy and protein-rich ingredients. This is achieved by enhancing low-quality feeds and employing strategies such as enriched feed blocks. However, the feeding practices and utilization of sheep and goats are influenced by various factors, including climate, breed, and production system, all of which are critical to consider when assessing their feeding needs. For example, animals that are mobile, such as those in nomadic or grazing systems, expend considerable energy on movement, which requires supplementation. Additionally, an animal’s body size, shaped by genetics, affects chewing rate, rumen pH, and bacterial composition. Meat production factors may be improved with proper use of NFs in small ruminants. This review explores the dietary needs of sheep and goats in relation to different production factors.

## Simple Summary

Focusing on meat production in sheep and goats in developing regions, nutrient utilization and requirements were revisited and discussed for the purposes of improvement in production efficiency in small ruminants commonly raised on nonconventional and lower quality feeds. The use of supplementation technology to alleviate both nutrient deficiencies and toxicity effects in small ruminants on lower quality diets appear to be viable and effective.

## 1. Introduction

Sheep and goats play a vital role as significant animals, serving essential economic and social functions worldwide, while improving living standards and alleviating poverty in rural areas. These animals are relatively easy to manage and can produce high-quality protein at a low cost, particularly when utilizing low-quality feed sources. Their meat is well-known for its tenderness and flavor, making it a popular choice among consumers. Both sheep and goats are capable of thriving in harsh environments, thanks to their physiological, metabolic, and molecular adaptation strategies.

Feed constitutes a major expense in sheep and goat production, accounting for 50–80% of total costs [1,2] depending on the rearing system. As a result, farmers frequently seek more economical feed options that may not be the best for the animals’ health and productivity. Understanding how sheep and goats metabolize nutrients can improve farming efficiency, enhance resource use, and reduce the carbon footprint. Proper nutrient utilization is vital for boosting meat production in sheep and goats, especially when they are given lower-quality feed.

Nutrient requirements are crucial factors to consider for precision nutrition in sheep and goat farming. These needs are influenced by various elements such as individual characteristics, genetics, environmental conditions, nutrition, age, and physiological stages. Gaining insights into specific nutrient requirements, enhancing feed management strategies. Generally, the dietary needs of goats are seen as similar to those of sheep. However, goats present unique benefits, including higher dry matter (DM) intake, lower water needs, and a superior capacity to digest crude fiber, enabling them to flourish in difficult environments [3]. Goats have been recognized for their distinct dietary preferences and digestive systems when compared to other domesticated species like cattle and sheep, showing more similarities to deer [4].

Sheep and goats are raised in diverse production systems, which include free-ranging, migratory, stall feeding, or a mix of these methods. Typically, they are maintained under suboptimal nutritional conditions, often relying on low-quality feeds supplemented occasionally, which affects their health and productivity. As a result, it is essential to consider their nutrient needs and utilization in relation to various factors, including production systems, climatic conditions, particularly high temperature and the quality of feed.

The aim of this review is to explore the intricacies of nutrient needs and their utilization in sheep and goats when fed low-quality feeds under different systems and climates. This provides important insights for enhancing meat production efficiency. As the agricultural industry evolves, it will be crucial to tackle the challenges and opportunities associated with providing novel feeds (NFs) to satisfy the increasing demand for high-quality meat products. By implementing innovative strategies and keeping abreast of advancements in nutritional research, we can establish a sustainable and prosperous future in sheep and goat farming.

## 2. Nutrient Utilization in Sheep and Goats

Nutrient utilization refers to the mechanism through which animals convert feed into body tissues, milk, and wool, which is vital for their health, growth, and overall productivity. Poor utilization can hinder their capacity for optimal production. Effective nutrient usage enables sheep and goats to transform their feed into high-quality products by improving the nutritional value of their diet and converting it into energy for growth and productivity. Several factors affect how sheep and goats utilize nutrients, such as the type of feed, the amount consumed, gut health, metabolic processes and the environment.

To optimize the production of sheep and goats, it is essential to enhance nutrient efficiency. A variety of strategies can be implemented to improve nutrient utilization in these animals. Dietary management practices aimed at maximizing nutrient use require precise selection of nutrient ratios, which can significantly affect outcomes. Customizing the diet to fulfill their specific needs and including high-quality ingredients will effectively improve nutrient absorption and utilization. Ben Salem [5] highlighted methods to enhance small ruminant production systems, which often face considerable nutritional deficiencies, leading to decreased productivity and reproductive success. These methods encompass: innovative technologies designed to increase feed resource availability, rumen modulation through natural substances to enhance microbial activity, improving diet quality, reducing feeding costs, and optimizing the management of livestock water supply.

Sheep and goats that consume low-quality feeds need supplementation to improve their production capacity and to tackle nutrient deficiencies and toxicity problems. Supplementation options include cereal grains and their by-products, leguminous forages, energy sources such as molasses, oilseeds, protein meals, as well as minerals and vitamins. Technical strategies for addressing specific nutritional challenges, such as the use of fodder trees, shrubs, and techniques like ensiling and feed blocks, can enhance the effective utilization of various agro-industrial by-products [5]. Collaborative initiatives should engage rural communities, agricultural researchers, and extension specialists to create and promote technologies and policies that can support sustainable livelihoods and boost the productive potential of drylands [6]. The combined application of economic energy sources, such as molasses, which provides rapidly fermentable carbohydrates, and urea, a non-protein nitrogen source, can facilitate the growth of rumen microbes and aid in the digestion of fibrous feeds [7].

## 3. Sheep and Goat Nutrient Requirements

Sheep and goats need a well-rounded diet that fulfills their specific nutritional requirements to promote their growth, reproduction, and overall well-being. Key nutrients are vital for their development, productivity, and reproductive success. These nutrients encompass: energy; protein; vitamins; minerals; and water. The fundamental aspects of this critical scientific knowledge involve understanding the needs and utilization of both structural and non-structural carbohydrates, degradable and bypass proteins, lipids, minerals, and vitamins. It may be necessary to adjust their diets, especially when sourced from NFs, to guarantee that sheep and goats receive the right balance of nutrients essential for their health and productivity.

A deeper comprehension of the nutritional needs of animals, rather than merely focusing on metabolic energy when broadly understood and applied in feeding strategies, could significantly impact the productivity of ruminants that currently rely on low-quality forages in tropical regions [8]. Information regarding metabolizable energy (ME) and crude protein (CP) content in feed has limited significance concerning how animals digest their feed without assessments in the rumen and within the animal itself. Consequently, it was suggested that this traditional approach is inadequate for forage diets in tropical settings [8]. Forecasting production levels should take into account digestibility, protein availability in the intestines, nutrient utilization efficiency, the animal’s physiological condition, and its prior dietary and health background.

### 3.1. Energy Requirements

Energy serves as the main limiting factor in the nutrition of small ruminants, and the energy level in their diet greatly affects feed efficiency and overall performance. A deficiency in energy can result in decreased output, reproductive challenges, increased mortality rates, and heightened susceptibility to diseases and parasites. Elevating dietary energy levels improves growth performance, nutrient digestibility, rumen fermentation, and barrier function, while also changing the composition of rumen bacteria.

The main energy sources for small ruminants consist of grains, pastures, browses, hay, fibrous by-products, and fat sources. Generally, the energy content in feed comes from the digestion of carbohydrates, fats, and proteins. Sheep and goats often experience undernourishment due to low-quality pastures and roughages or inadequate feed, resulting in energy deficiency.

The main energy source for ruminants is fiber, which undergoes fermentation in the rumen. The end products of microbial fermentation in the rumen, volatile fatty acids (VFA), are absorbed through the rumen wall and utilized for biochemical synthesis, producing substances that can serve as energy sources, be stored as body fat, or be transformed into milk fat [9]. Forage-based diets rich in fiber yield higher amounts of acetic, butyric, and isobutyric acids, while concentrate diets lead to a larger production of propionic acid [9]. The quality of the feed significantly impacts its utilization by small ruminants. Goats that were fed high-fiber diets showed lower peak plasma glucose levels [10], indicating that less glucose was used for energy and more was stored as milk fat.

Increased energy levels affect the feed intake in animals. Overall, it has been noted that the ideal dietary ME for sheep during the growth phase ranges from 9.8 to 10.4 MJ/kg [11]. When it comes to raising goats, a dietary ME density that surpasses 11.63 MJ/kg can reduce their intake and hinder their growth rate [9].

There are differences in views in the way animals utilize dietary nutrients for tissue development and whether they can be affected by factors such as dietary energy levels, genotype, and environmental conditions. For instance, Trenkle [12] noted that in cattle that were slaughtered at similar body weights and genotypes, the energy levels in their diets did not affect the makeup of their body tissues. Conversely, Ferrell et al. [13] found out that diets high in concentrates could lead to a marginally increased fat content in empty body tissues compared to low-energy diets. Similar observations have been made in sheep [14]. The production levels achieved on forage-based diets, where ruminants receive supplements to optimize feed efficiency, may not be greatly influenced by the genetic growth potential of the animal [8].

Enhancing meat production from sheep can be achieved through greater body weight (BW) gains and improved carcass composition by elevating energy levels in their diet. Mahgoub et al. [15] indicated that the digestibility of dry matter (DM) increased as energy levels in diets rose. An increase in ME density led to higher daily BW gain and a better feed conversion ratio. Sheep that were given a high-energy diet exhibited greater BW, empty body weight (EBW), and carcass weight, along with a higher dressing percentage, but had lower gut content compared to lambs on medium and low-energy diets. Sheep that were slaughtered at the conclusion of the experiment showed reduced water and protein content yet had increased carcass and non-carcass chemical fat [15].

Increasing the concentration of dietary energy led to a gradual rise in empty body weight (EBW), as well as in the water, protein, and fat content of both carcass and non-carcass components. Additionally, higher dietary energy concentrations resulted in an increased accumulation of energy in carcass fat while decreasing the energy stored in carcass protein [16].

The concentration of dietary energy did not influence the energy distribution between protein and fat in empty body and non-carcass tissues. Furthermore, the ratios of energy to empty body, carcass, or non-carcass weight remained unchanged despite variations in dietary energy concentrations [17]. A greater amount of energy is necessary for the deposition of carcass tissues compared to non-carcass tissues. Moreover, dietary energy concentration did not impact nitrogen (N) digestibility or retention. However, animals on a low-energy diet retained significantly less nitrogen in their empty bodies than those on a high-energy diet [17].

### 3.2. Protein Requirements

Protein plays a crucial role in various bodily functions, including maintenance, growth, reproduction, milk production, fiber synthesis, and immune response. The protein requirements differ depending on the production stage (such as growth, pregnancy, or nursing) and particular health concerns (like internal parasites or dental issues). For small ruminants, the amount of protein is generally more important than its quality due to the processes of microbial fermentation and modification occurring in the rumen. When the primary goal is to supplement protein, particularly when feeding low-quality feeds, the cost of protein becomes a significant consideration.

Both feed intake and digestion decline when the raw protein content in the diet falls below 6%, exacerbating protein deficiency. Therefore, healthy adult animals should have a diet that includes at least 7% crude protein (CP) [18]. Most sheep need a minimum of 7% CP in their diet to sustain their condition [19]. The requirements for dietary crude protein increase during periods of growth, gestation, and lactation [18]. The digestible CP needs for maintenance in sheep and goats range from 2.3 to 2.8 g/kg 0.75, but these requirements can rise by 80–100% during the later stages of pregnancy [20]. To fulfill their maintenance requirements, sheep should consume forage with a crude protein content of 7 to 9% and a total digestible nutrients (TDN) value of 50% [20]. These values increase under different physiological conditions and heightened production demands. If the dietary CP is below 6%, both feed intake and nutrient digestibility decrease, leading to a more severe energy-protein deficiency; thus, to keep mature, healthy animals, the diet must include at least 7% crude protein.

Supplementing a low-protein diet is crucial and is typically achieved by in ruminants using urea as a non-protein nitrogen (NPN) source. Urea serves as an economical NPN source frequently employed in ruminant nutrition. It is particularly recommended for sheep and goats that are provided with low-quality forages. Within the rumen, microorganisms convert urea into microbial protein that the animal can assimilate. A common practice with small ruminants involves enhancing low-quality hay with molasses blocks that contain varying amounts of urea. For instance, providing complete feed blocks that include rumen-protected fat and urea as a cost-effective N source has been shown to enhance animal performance, resulting in improved nutrient intake, utilization, and efficiency, along with better carcass traits [20].

### 3.3. Mineral Requirements

Minerals are essential for the health and productivity of livestock. Adequate mineral intake improves reproductive success and growth in animals. Different animal species and production levels have specific mineral requirements. The primary sources of minerals are diet, various mineral supplements, and, in some areas, the water supply. For animals consuming low-quality feeds, it is especially important to address deficiencies and keep track of excess intake to prevent toxicity.

Overall, the mineral needs of goats are comparable to those of sheep. Goats and sheep need 23 vital minerals, which include both major and trace elements. The major minerals consist of Calcium (Ca), Phosphorus (P), Magnesium (Mg), Potassium (K), Sodium (Na), Chlorine (Cl), and Sulphur (S). The trace minerals encompass Iron (Fe), Iodine (I), Copper (Cu), Manganese (Mn), Zinc, Cobalt (Co), Molybdenum (Mo), Selenium (Se), Chromium (Cr), Tin (Sn), Vanadium (V), Fluorine (F), Silicon (Si), Nickel (Ni), Arsenic (As), and Lead (Pb) [21]. A deficiency in these minerals can result in metabolic disorders, which can be avoided or addressed by supplying the lacking minerals. Nevertheless, goats possess a limited capacity to adjust to low mineral levels, such as magnesium, by reducing the amount of magnesium they excrete; both urinary excretion and milk production decline in cases of magnesium deficiency [18].

Mineral supplements are crucial for sheep and goats that graze pastures. Although the grass may appear lush and green, the soil can have inherent deficiencies or excesses, and if certain nutrients are not available, the forage will not meet their needs. Minerals can be added to their diet or made available as a free-choice option for undernourished sheep and goats. Research indicates that sheep can distinguish between different flavored feeds containing Salt (NaCl), calcium carbonate (CaCO_3_), and dicalcium phosphate (CaHPO_4_), showing preferences that reflect the mineral deficiencies in their usual diets [22]. Consequently, it may be practical to offer Ca and P supplements on a free-choice basis, enabling individual animals within a group to express preferences based on their specific needs. Nonetheless, it is advisable that, whenever feasible, essential minerals should be incorporated into the diet instead of being provided on a free-choice basis [23].

Forages are generally abundant in various minerals, such as potassium, which makes deficiencies in grazing goats quite rare, except for heavily lactating does that primarily consume cereal grains [24]. However, when sheep and goats ingest low-quality NFs, they may face mineral deficiencies, making supplementation highly recommended. The amount of calcium retained in the weight gain of lambs was 11.00 g/kg of EBW gain, while the net calcium needed for sheep maintenance was 0.016 g/kg of body weight (BW), as defined by ARC [24]. Diets containing Ca levels between 0.73% and 0.89% can improve growth performance and calcium utilization efficiency of rams [25]. Ca and P are interrelated. Therefore, an adequate supply of both is crucial, and they should be included in the diet in appropriate ratios. The Ca:P ratio in goats should be maintained between 1:1 and 2:1, with an ideal range of 1.2–1.5:1, due to their vulnerability to urinary calculi. Grazing goats are more prone to P deficiency compared to Ca deficiency. It is common practice to provide small ruminants with Ca and P sources, such as dicalcium phosphate. An increased intake of CaHPO4 results in higher fecal losses of both Ca and P, thereby diminishing the efficiency of utilization for these minerals [26]. Additionally, elevated dietary P levels have been associated with increased endogenous P excretion, contributing to the overall P released into the environment. Excess Na in the diet has been shown to impair microbial protein synthesis in the sheep rumen and reduce the apparent digestibility of total nitrogen in the small intestine [27].

Sheep and goats may be raised on high salt lands such as salt bush. A major limitation of these pasture systems is the high NaCl content of the forage and its physiological implications for grazing animals [28]. Incorporating low NaCl alternatives has been found to improve livestock performance from high NaCl feeds. Sheep actively choose combinations of high NaCl and alternative feeds that enhance the overall nutritional value of their diet [29]. Sheep provided with a low-energy alternative feed exhibited a greater intake of the high NaCl feed compared to those given high-energy alternatives. Nevertheless, the intake of the high NaCl feed did not show a significant difference in sheep given a low CP alternative versus those given a high CP alternative feed. Sheep initially selected a mix of high and low NaCl feeds that remained unchanged as they became accustomed to the feeds. The total organic matter intake (OMI) of sheep offered any of four low NaCl alternatives was approximately 50% higher, and their liveweight gain (LWG) was at least double that of sheep that were only given the high NaCl feed [29].

Sulfur (S) is an essential mineral crucial for animal nutrition, particularly for ruminants, where it plays a significant role in various amino acids, vitamins, and enzymes. It is vital for microbial protein synthesis in the rumen, aiding in the production of biotin, thiamine, and collagen, while helping to maintain blood pH and facilitating calcium utilization. Adequate S intake is critical for overall animal health, growth, and productivity, including wool and mohair production in sheep and goats. However, excessive intake can result in toxicity, making it necessary to monitor S levels in animal feeds. Excellent sources of S include legumes and brassicas; forages from plants grown in S-rich soils; oilseeds such as soybean meal; and byproducts from molasses and sugar beet processing. In some regions, water sources can also contribute a considerable amount of S, especially in the form of sulfates. S can be supplemented through feed blocks, mineral mixes, or other formulations. The S needs for goats can be divided into two primary components: inorganic sulfur and sulfur-containing amino acids [30]. The inclusion of dietary S resulted in an increase in urinary S output, sulfate concentrations in feces, and also improved S absorption and net retention. The levels of serum Cu and Zn were unaffected by S supplementation. An increase in S intake led to a higher concentration of protein S in the rumen, better apparent absorption of Zn, and improved Zn retention [30]. Additionally, urinary Zn excretion increased with higher S levels. In contrast, urinary Cu output decreased, and the metabolism of Mo remained stable due to S supplementation [31]. The addition of sulfate enhanced average daily gain (ADG) and dry matter intake (DMI) in goats, along with a rise in urinary uric acid output, suggesting improved ruminal bacterial protein synthesis. The optimal dietary sulfur levels for achieving maximum ADG, DMI, nitrogen retention, and absorbed nitrogen retention was estimated at 0.22%; 0.24%; 23%; 22% [32].

Iron is a vital element for livestock. A deficiency in iron can lead to anemia, which is characterized by a lack of red blood cells, hemoglobin, or both. Access to pasture or high-quality trace mineral salt that includes Fe can help prevent its deficiency. Kids and lambs raised indoors on maternal milk exhibited notably lower hemoglobin levels compared to those fed milk replacer or those raised outdoors [33]. Administering Fe dextran to lambs and kids resulted in elevated hemoglobin levels at one month of age and significantly improved growth rates, particularly in twin lambs [33].

Iodine is an essential trace element for animals and is frequently added to animal feed due to its scarcity in the natural diets of many species, typically supplied in stabilized salt. Supplementation of I has been shown to restore fertility in sheep residing in areas deficient in I and may serve as a method for achieving I prophylaxis in local sheep [34]. Goats are particularly at risk due to their browsing habits and potential limited access to I-rich foods, or by consuming plants such as cabbage, kale, broccoli, cauliflower, and turnips, which may contain goitrogenic compounds that hinder I absorption. The I-deficiency can be accurately evaluated by measuring I levels in milk and urine. Daily I supplementation can avert deficiency. It is crucial to avoid feeding goats diets high in goitrogens, as these compounds can interfere with I utilization and exacerbate deficiency symptoms [35].

Zn is a crucial trace element that is essential for numerous physiological functions such as growth, appetite regulation, immune response, skin and skeletal health, and reproduction. Diets lacking sufficient Zn can lead to a higher occurrence of abortions and stillbirths. Zn plays a role in the activity of over 300 enzymes and hormones. Typically, it is added to animal diets in low amounts, usually less than 200 mg per kg in complete feeds. To avoid deficiencies, a minimum of 10 ppm of Zn in the diet or a trace mineral salt mixture containing 0.5%–2% Zn is recommended. Excessive calcium intake from alfalfa may heighten the risk of Zn deficiency in goats. Underwood and Somers [36] reported that ram lambs fed a diet containing 2.4 ppm of Zn showed stunted growth, exhibited clinical symptoms of Zn deficiency, experienced diminished testicular development, and underwent a complete cessation of spermatogenesis over a duration of 20–40 weeks. Conversely, lambs receiving the same diet supplemented with Zn sulfate to reach total Zn levels of 17.4 and 32.4 ppm demonstrated increased feed consumption, achieved greater LWG, and showed no signs of Zn deficiency. Both Zn supplements promoted testicular growth and enhanced sperm production. All signs of Zn deficiency were entirely resolved, and there was a complete restoration of testicular size, structure, and function during a Zn repletion phase lasting 20 weeks [36].

Se is a crucial trace element for animals, significantly contributing to their growth, reproduction, and immune functions. A deficiency of Se in the diet is often linked to nutritional muscular dystrophy, retained placentas, metritis, poor growth, weak or premature offspring, and mastitis. Conversely, Se toxicity can arise when animals ingest excessive amounts of Se, whether through chronic or acute exposure. Acute toxicity may result from an overdose of Se supplements, while chronic toxicity typically stems from a prolonged high-Se diet [18]. Nevertheless, the combined effects of Se and vitamin E on reproductive outcomes were significant, as goats fed diets containing 0.5 mg/kg Se and 20 mg/kg vitamin E produced heavier offspring compared to those on alternative dietary regimens. Both organic and inorganic Se supplementation improved growth rates, humoral immune responses, and antioxidant levels in lambs; furthermore, organic Se was found to be more effective than its inorganic counterpart among the two sources [37].

Cu is an essential trace element for animals, contributing to various physiological functions including the development of bones and joints, the proper functioning of the digestive tract; it aids in forming a protective sheath around nerves; it assists with hair pigmentation; and it plays a role in hemoglobin formation while being involved in numerous enzyme activities. A deficiency of Cu in the diet can arise from insufficient Cu intake, a reduced Cu-Mo ratio, or excessive dietary S. This deficiency may lead to microcytic anemia, decreased production, lighter or faded hair color, poor fiber quality, infertility, compromised health, and stunted growth, as well as certain types of metabolic bone disease, diarrhea, and potentially increased vulnerability to internal parasites. The utilization of Cu by small ruminants is influenced by several factors. For example, the consumption of sulfate and Mo leads to the creation of thiomolybdates, which have a strong affinity for Cu, making it unavailable for absorption [38,39]. Nevertheless, excessive Cu can also be detrimental, although goats seem to exhibit greater resistance to Cu toxicity compared to sheep. Given that sheep are susceptible to Cu toxicity, a general livestock mineral mix that does not contain Cu is typically provided to the group [40].

Mineral needs must be evaluated in the context of feed quality, climate and environment, production systems, and genetics. Teixeira et al. [41] indicated that the P, Na, and K requirements of goats in hot climates vary from those in other feeding systems and climates, likely due to the adaptation strategies that goats utilize to manage high temperatures. It is possible that goats allocate energy and nutrients to handle heat stress and other challenges linked to hot environments.

Integrating specific minerals (such as P for dry winter forages and Se in areas with low supply) into NaCl, preferably in granule form and provided ad libitum, helps prevent various mineral deficiencies and improves overall performance. In grazing scenarios, meat-type goats generally meet their calcium requirements more effectively than dairy goats. For goats that browse or are fed grains, adding a Ca supplement (like Ca hydrogen phosphate or limestone) to their feed or mixing it with NaCl or trace mineral salt blend usually satisfies their Ca needs.

### 3.4. Vitamin’s Requirements

Vitamins play a crucial role in growth, health, and reproduction of animals. Small ruminants require a diverse range of vitamins. However, their vitamin requirements are relatively simple because of the feed they usually consume and the vitamins produced in the rumen. It is important to take this into account when offering low-quality diets such as NFs, as these may be deficient in essential vitamins that must be supplemented.

Vitamin A is produced in the bodies of sheep and goats from beta-carotene found in green plants and is stored in the liver [42]. Therefore, they are unlikely to suffer from a vitamin A deficiency unless they are deprived of beta-carotene for a prolonged period, similar to stall-fed animals on NFs. Unlike humans, ruminant animals do not need B vitamins in their diet because the microorganisms in the rumen can synthesize all essential B vitamins. However, if goats with a digestive problem such as acidosis from consuming too many concentrates, the beneficial microorganisms that generate Thiamine (Vitamin B1) may be lost. Vitamin C is essential for the immune system to function properly. While most animals can produce their own vitamin C, sheep and goats still need it in their diet. A vitamin C powder that dissolves in water can be mixed into drinking water for sheep and goats. Vitamin D is produced in the skin of goats when they are exposed to sunlight. It should be part of the diet for goats kept indoors. Fresh, sun-dried hay serves as an excellent source of vitamin D.

Vitamin E works in conjunction with the mineral Se to support healthy growth. The term “white muscle” disease describes the breakdown of muscle tissue affecting young goats. Treatment with vitamin E and Se can aid in preventing or improving this condition. Vitamin K is synthesized by microbes in the rumen and is also plentiful in various feeds.

However, most supplementation decisions in sheep production systems are made without veterinary advice or laboratory evidence, resulting in a lack of an evidence-based approach [43]. Knowledge transfer activities need to be organized to effectively communicate best practices for supplementation. Typically, a free-choice salt-vitamin-mineral premix should be available to small ruminants at all times, unless a premix is already included in the grain ration or total mixed ration.

## 4. Factors Affecting Nutritional Needs in Small Ruminants

Dietary needs are influenced by factors such as body size, genetic composition, climate, growth rate, and physiological condition, along with the interactions among these elements. It is essential to consider these factors when providing low-quality diets to animals. The environment significantly impacts animal nutrition, especially due to how ambient temperature affects feed intake and its utilization. Rearing systems include components like animal movement, which influences energy expenditure [44]. The genetics of the animals relate to variations in body weight, which in turn affect feed intake and utilization based on the amount of feed consumed, chewing, and overall efficiency in utilization.

### 4.1. The Environment

The environment can influence the utilization of dietary nutrients for tissue development. A significant factor in this context is the surrounding temperature where animals reside. To sustain normal metabolic functions, environmental stressors like extreme temperatures heighten the demand for maintenance. For example, sheep that graze in warmer regions require additional energy to manage their body temperature, leading to increased energy expenditure. As the ambient temperature exceeds 25–27 °C, more energy is consumed to maintain core body temperature [28]. Consequently, the utilization of dietary nutrients for tissue growth may be affected. It is essential to evaluate how climate influences the nutritional needs and feed intake when utilizing low-quality feeds for the maintenance and production of sheep and goats. In comparison to other sheep breeds in temperate regions, as established by the US National Research Council (NRC) [28], Agricultural Research Council (ARC) [24], and the UK Agricultural and Food Research Council (AFRC) [45] for male lamb production, Omani sheep in hotter climates exhibit higher Net Energy of Gain (NEg) requirements, and thus, greater ME for Gain (MEg) requirements [16].

Salah et al. [46] conducted a meta-analysis of 590 publications to update the energy and protein requirements based on metabolic live weight (MLW) for growing sheep, goats, and cattle in warm regions. The energy maintenance requirements for sheep and goats were found to be 542.64 kJ ME/kg LW^0.75^, while the growth requirement was 24.3 kJ ME/g BWG. There was no difference in ME requirements for maintenance and gain among different genotypes relative to LW^0.75^. However, it was noted that small ruminants in warm and tropical climates exhibited higher ME maintenance requirements compared to those in temperate climates and cattle. The analysis of digestible protein intake (DPI, g/kg LW^0.75^) in relation to retained nitrogen (RN) (g/kg LW^0.75^) revealed that the DP requirements for sheep are significantly greater than those for goats, with no notable difference in the amount of DPI per gram of retained crude protein (RCP). Furthermore, regressing metabolizable protein (MP) or minimal digestible protein in the intestine (DPmin) against RCP indicated no differences between species or genotypes, both in terms of the intercept (maintenance = 3.51 g/kg LW^0.75^ for sheep and goats) and the slope (growth = 0.60 g MP/g RCP). The regression of digestible crude protein (DCP) against average daily gain (ADG) demonstrated that DP requirements were consistent across species and genotypes [46].

### 4.2. Rearing Systems

An important aspect to consider about energy use in small ruminants is the management system that is implemented. Salah et al. [46] revised the energy and protein needs of sheep and goats raised in various systems. These systems encompass migratory (nomadic and transhumant), stall feeding, free-ranging, and a mix of these methods. Increased activity levels, such as those observed when sheep or goats roam large areas in search of food and water, lead to higher maintenance requirements for these animals compared to those housed in feedlots or stalls. In free-ranging environments, animals cover vast distances to meet their energy needs, which can significantly influence the maintenance requirements for ME. The energy demands in such a system may rise considerably when compared to estimates for animals kept in confinement. The nutrient and energy need of animals foraging on semi-arid highland pastures are substantial, as they travel long distances in search of food [47,48]. However, small ruminants typically demonstrate an impressive ability to thrive in arid climates and can secure sufficient nutrition even when forage is limited [2]. The native plant life in dry and semi-dry mountainous areas exhibits less than ideal nutritional quality, which in turn restricts the productivity of grazing livestock [6,49] and thus requires a sufficient supply of energy and nutrients. Reduced levels of CP in rangeland vegetation may hinder the digestibility of the diets ingested by grazing ruminants. Consequently, supplying a protein-rich concentrate mixture can greatly enhance the total DMI of these animals [6].

Energy expenditure in goats affects their nutritional requirements, especially when their nutrition levels are inadequate, and it varies based on the rearing method. Since pasture animals encounter a variety of environmental factors such as temperature, humidity, solar radiation, walking distance, and pasture availability, their energy expenditure fluctuates significantly according to the landscape. In semi-arid areas, sheep use 43% more energy grazing on grass compared to being stall-fed [50]. Sheep grazing in pastures have a maintenance energy requirement that is 25–100% greater than that of animals raised in confinement. The estimated maintenance energy needs for sheep range from 389 to 460 KJ/kg W^0.75^, while for goats, they are between 424 and 522 KJ/kg W^0.75^ [51].

The National Research Council (NRC) [28] suggested increasing energy requirements by 25% for animals with light activity, 50% for those in semiarid rangeland and slightly hilly terrain, and 75% for long-distance grazing, as measured by indirect calorimeters [52,53]. According to NRC [28], goats show increased muscle activity while grazing. This leads to a 25% rise in energy needs for light activity, a 50% rise for semi-arid rangeland pastures and slightly hilly regions, and a 75% rise for long-distance travel across sparsely vegetated grasslands or mountainous transhumance pastures [2]. The additional energy required for feeding and the energy expended during walking contribute to this increased maintenance requirement. Energy expenditure estimates for walking were 3.35, 31.7, and −13.2 J kg^−1^ BWm^−1^ for horizontal, upward, and downward movements, respectively [2]. Heat production (HP) for confined goats reached 401 kJ kg^−0.75^ per day. The increase in HP for unrestrained goats compared to restrained ones was 43.1%, with the daily distance traveled (approximately 2 km) accounting for 9% of the extra HP. It has been suggested that the energy expenditure for activity (MEa) in stall-fed goats constitutes 10% of their fasting heat production (10% of 315 kJ/kgBW^0.75^), in addition to extra costs for grazing calculated on a body weight basis. This includes horizontal movement (3.5 J/(kgBW × m)), vertical movement (28 J/(kgBW × m)), standing (0.417 kJ/(kgBW × h)), and position changes (0.26 kJ/(kgBW × number of changes)), along with the efficiency of ME utilization for maintenance [2]. In this regard, it is wise to assess the nutritional requirements of migratory herds of sheep and goats that engage in an annual cycle of ascending (4000–5000 m above sea level) and descending migration (to plains) and face varying degrees of undernutrition [2].

Dickhoefer et al. [54] observed that the pasture vegetation in Jabal Akhdar, Oman, exhibited minimal ME (7.2 MJ/kg OM) and P levels (1.4 g/kg OM). Providing grazing goats with nutrient-rich and energy-dense by-products from the national fishery and date palm agriculture improved their daily OM intake and satisfied their growth and production requirements. While the OM intake from pasture was highest in animals on a concentrate-based diet, the consumption of 21 g OM/kgW^0.75^ of cultivated green fodder led to a reduction in the animals’ pasture feed intake. Adjusting homestead supplementation with locally sourced feed to cater to the specific needs of individual goats and the nutritional quality of pasture plants boosts animal performance and alleviates grazing pressure on native vegetation [54]. As a result, some researchers have proposed a shift from pastoral systems to zero-grazing systems to improve animal production and protect the natural rangeland vegetation [55,56]. Available local feed resources can be utilized for this purpose. For example, Mahgoub et al. [57] suggested that date palm by-products could be employed to feed Omani sheep for maintenance or during periods of nutritional scarcity often encountered in the arid tropics.

### 4.3. Genetics

The breed of sheep and goats can influence their nutritional requirements, as it affects their body size and composition, along with their level of adaptation to the environment. The breed of sheep can greatly affect the distribution of chemical components between the carcass and non-carcass tissues [58]. Compared to lighter ones, breeds of sheep and goats that are larger and grow more quickly generally have a higher proportion of their empty body weight and chemical components in the carcass [59]. However, smaller breeds may utilize nutrients for meat production more efficiently than their larger counterparts [60]. It is estimated that sheep and goats consume between 2% and 4% of their body weight when calculating feed intake on a dry matter basis [60]. Nevertheless, this percentage can differ depending on the size of the animal, as smaller animals usually need a higher intake percentage to sustain their weight [61].

### 4.4. Physiological Status

The nutritional needs of sheep and goats increase during their development, lactation, and pregnancy. While sex and genotype influence the efficiency of energy utilization for growth, but do not affect the efficiency of protein use. There was is no effect of the duration of pregnancy on energy or protein requirements [41]. The efficiency of ME use during pregnancy improves as pregnancy advances.

Table 1 compiles the nutrient requirements for sheep and goats at various life stages, as described by the NRC [28,61].

The nutritional needs for native Indian lambs to achieve a higher growth rate were calculated at 9.50 MJ ME and 9.04% DP per kg of diet, aiming for an ADG of 124 g with a feed conversion ratio of 6.92 [60]. Paul et al. [60] employed regression analysis to ascertain the necessary amounts of TDN, CP, and DP for maintenance and body weight gain (BWG) by analyzing data from 19 feeding trials conducted on growing sheep across various institutes in India (Table 2). Additionally, Mandal et al. [62] estimated the TDN, CP, and DP requirements for maintenance from twenty-five feeding trials involving growing goats, which were found to be 30.1, 5.83, and 3.22 g/kg BW^0.75^, respectively, while the requirements for gain (g/day) were 1.61, 0.45, and 0.34 g. A recommendation of 32–35 g DCP/Mcal ME was made for achieving an ADG between 130 and 160 g [63]. The DMI and nutrient utilization were similar in both mutton synthetic and selected Malpura lambs, requiring 75 g DM, 5.5 g DCP, and 42.1 g TDN/kg W^0.75^ to achieve a daily gain of 148 g [64].

It is important to recognize that significant variations exist in energy estimation across various animal breeds and classes, as highlighted in reputable sources such as NRC and ARC. For example, Lu and Potchoiba [66] observed that the ME requirement for growth was 33% less than the figure suggested by the NRC in 1981 [28].

### 4.5. Feeding Novel Feeds for Sheep and Goats

Novel feed (NF) sources that have not been traditionally used for animal nutrition by either farmers or feed manufacturers in commercial feeds are widely employed for livestock feeding [67]. NFs are utilized in specific proportions depending on their palatability, nutritional advantages, and the presence of toxic or anti-nutritional factors. Most of these feeds are noted for their low energy, protein, and mineral content, alongside high fiber levels, moderate to low digestibility, and the presence of contaminants. They can be classified as energy sources, protein sources, or miscellaneous sources. Furthermore, they can be divided into categories such as browse leaves, concentrates, crop residues, agro-industrial by-products, and other feed types. In general, these diets fail to supply the necessary components for growth and production, which negatively affects animal performance and influences the efficiency of meat quality. With appropriate precautions and treatments, they can be effectively incorporated into the diets of small ruminants up to a certain inclusion level [68].

A significant drawback of using NFs is the presence of antinutritional factors that interfere with normal digestion, absorption, and metabolism, with some possibly leading to negative impacts on the animal’s digestive system. To improve the nutritional advantages of these feeds, they are treated with specific additives or shaped into feed blocks. The effectiveness of NFs is determined by the nutrient concentration they possess, especially protein. Typically, they require additional key nutrients, such as vitamins and minerals, to address deficiencies, thereby allowing them to support production adequately. For example, urea is a cost-effective NPN source commonly incorporated into the diets of ruminants.

### 4.6. Utilization of Browse Species by Sheep and Goats

Trees and shrubs are often used to feed small ruminants, especially in tropical regions, and can help reducing costs associated with meat and milk production. Although they frequently contain valuable nutraceuticals, proteins, and other nutrients particularly in their leaves, their use is limited due to low palatability and the presence of anti-nutritional factors [69]. Many tropical browse species have high levels of phenolic compounds, primarily tannins [70]. There are differences in how sheep and goats utilize browsing species. The dry matter (DM) intake per unit of body weight was almost 50% higher in goats compared to sheep that were fed *Prosopis cineraria* leaves [71]. The low DC percentage in both sheep and goats may be attributed to the formation of an insoluble and indigestible tannin-protein complex within the intestines of the animals. The poor digestibility of Acid Detergent Fibre (ADF) and lignin is likely due to the formation of specific compounds in the hindgut of the animals. Goats exhibited higher digestible energy (DE) and ME from the feed, along with better digestibility coefficients for various cell-wall components and proteins, compared to sheep. Additionally, intake and digestibility were greater for goats consuming browse species [71]. Goats were more efficient in digesting nitrogen (N) than sheep, although there were no significant differences in DE and ME between the two species [72]. Nonetheless, it was observed that, in tropical environments, goats appear to be better suited for digesting tannin-rich diets than sheep [73].

Plants generate secondary compounds such as tannins, saponins, flavonoids, glucosinolates, mimosine, and essential oils, which can influence ruminal fermentation [74]. These compounds are frequently regarded as toxic due to their ability to inhibit microbial growth and/or diminish performance in host ruminants. Natural feed additives derived from these secondary compounds, have the potential to improve feed efficiency and enhance productivity in ruminant animals [75]. Secondary metabolites impact digestibility, dry matter consumption, and nutrient absorption in ruminant nutrition [74]. The dosage given determines whether these compounds are beneficial or detrimental. Key secondary metabolites like tannins, saponins, and essential oils can promote health, increase average weight gain, and enhance the production of milk and wool. Ruminant animals that consume forages high in secondary compounds exhibit more efficient energy utilization and lower methane (CH_4_) emissions due to decreased rumen gas production. An increased intake of tannins in the diet can result in reduced CH_4_ emissions when supplementing with tropical plants [76]. Nevertheless, a major obstacle in integrating such forages is the absence of a consistent recommended dosage [74]. Alfalfa saponins have been shown to decrease microbial protein synthesis in the rumen [77]. Leucaena mimosine is transformed into the toxic goitrogen 3-hydroxy-4(1H)-pyridone, leading to toxicosis in goats [78]. Milkvetch leaves contain arabinogalactan protein, which inhibits cellulolytic bacteria from adhering to cellulose [79].

Low molecular weight tannins found in leguminous plants such as *L. leucocephala* [80], *Calliandra calothyrsus* [81], and subterranean clover (*Trifolium subterraneum*) [82] do not impede protein digestibility, which could otherwise restrict ruminant feed intake and performance. Plants like *Allium sativum*, *Coriandrum sativum*, *Eucalyptus globulus*, *Foeniculum vulgare*, *Mentha piperita*, *Ocimum sanctum*, *Populus deltoides*, and *Syzygium aromaticum* are rich in essential oils that effectively suppress protozoal growth in the rumen. However, these may have a detrimental effect on feed degradability and nutrient absorption in ruminants [83]. Condensed tannins (CT) might improve intestinal digestibility and enhance energy utilization by reducing methane emissions from OM in the rumen. Nevertheless, Gemeda and Hassen [84] noted that tannins could negatively influence feed digestion and nutrient absorption in the small intestine.

CT has a detrimental effect on the performance and digestion of lambs [85]. However, the performance and meat quality of lambs were similar to those receiving a maize-based diet, as the inclusion of 40 g of polyethylene glycol (PEG) per kg of feed mitigated the adverse effects of CT. Lambs on a basal diet of hay, supplemented with *Albizia gummifera* (AG) leaves and PEG, exhibited higher feed intake, growth rates, and carcass weights, along with improved feed conversion efficiency, compared to those fed hay alone or hay with AG [86]. The apparent digestibility coefficients for all nutrient sources were greater in the AG-PEG and AG diets than in the hay-only diet [86].

### 4.7. Nitrogen Utilization in Small Ruminants Fed NFs

Diets of poor quality, such as NFs, often have insufficient protein levels that negatively affect the health and productivity of small ruminants, and this issue needs to be addressed. One approach to support small ruminants is to incorporate non-protein sources like urea as a dietary supplement. Goats that did not receive urea supplementation experienced a negative nitrogen balance, in contrast to those that consumed the 6% urea blocks, which exhibited a positive balance [87]. A minimum crude protein requirement of 8% for maintenance in goats fed low-quality forage, along with a urea-based supplement, has been established. Increasing the urea content in molasses blocks to 6% significantly improved nitrogen intake, retention, and balance in goats [87].

Although there is a reduction in DMI for goats compared to sheep, their superior digestive efficiency, especially regarding cell-wall components, results in a comparable nutritional status for both species [88]. The reduced levels of rumen NH3-N in goats suggest their effective use of dietary protein. The enhanced digestibility of cell-wall components in goats is associated with a greater population of rumen protozoa. Sheep have the ability to convert NPN (such as urea, ammonium phosphate, and biuret) into protein within the rumen. This nitrogen source can contribute at least partially to the required supplemental nitrogen in high-energy diets with a nitrogen-to-sulfur ratio of 10:1. In lamb-finishing diets, adding alfalfa, approved growth enhancers, and a source of fermentable carbohydrates (like ground corn or ground milo) improves nitrogen utilization [18].

Integrating protein and energy sources into low-quality feeds enhances the performance of small ruminants in meat production. Li et al. [89] examined the impact of various protein-to-fat ratios in feed components on the growth, slaughter performance, and meat quality of lambs. Lambs receiving a protein-to-fat ratio of 50:10 exhibited the greatest weight gain, feed conversion efficiency, and increased carcass weight and slaughter rate when compared to those on 50:5 and 50:20 protein-to-fat ratios. The feeding system influences protein utilization in sheep. A study comparing stall feeding to grazing with supplementation indicated that stall-fed animals had higher levels of total N and NH3-N than those that grazed with supplementation [90].

### 4.8. Energy Utilization in Sheep and Goats on NFs

Through the degradation of plant cell walls, thus reducing the breakdown of plant proteins as a gluconeogenic dietary fiber plays a crucial role in balancing nutrient needs and affects how goats interact with nutrient intake and digestion [9]. Both sheep and goats require adequate dietary fiber to maintain normal rumen function, which is linked to rumination, facilitating proper salivation and creating an optimal pH for cellulolytic microbes that typically yield higher acetate to propionate ratios in the rumen fluid [9]. However, low-quality feeds like NFs often have high fiber content, which can influence the animals’ feed intake and nutrient absorption. In goats consuming high-concentrate diets, the physiological regulation (feedback from metabolic parameters) of intake is the primary factor, whereas in those on high-forage diets, physical fill becomes the dominant factor. In contrast, in goats fed high-forage diets, physical fill is the dominant element in regulating intake [9]. The intake of dietary fiber impacts mastication and rumen fermentation by affecting buffering capacity and salivation. As fiber intake increases, so does the time spent eating, ruminating, and overall chewing [91,92].

#### 4.8.1. Effect of Feed Particle Size on Digestion and Nutrient Utilization in Small Ruminants

Low-quality forage that small ruminants consume typically has a high fiber content, which affects their capacity to reduce feed particle size through chewing. This is further complicated by factors such as body size and breed. A diet high in fiber changes the chewing rate, resulting in longer rumination times that may limit voluntary feed intake and disrupt normal fermentation processes in the rumen, as well as influence the production of volatile fatty acids [9]. Dairy goats fed hay with shorter particle lengths exhibited shorter eating and rumination durations compared to those given longer particle lengths [9]. Around 50% of the variations in chewing behavior can be linked directly to the size of the animal [93], which is influenced by several factors including breed, developmental stage, and nutrition. When metabolic size is taken into account, larger animals typically need less time to chew per unit of fiber consumed [94]. Chewing and rumination decrease particle size, enhancing the surface available for digestive microbes and enzymes in the rumen, and speed up the movement of digesta. The process of salivating during chewing affects the dilution rate and buffering capacity of rumen fluid.

The total chewing duration, expressed per unit of DM intake adjusted for metabolic body weight (MBW), showed a linear increase with dietary ADF intake [91]. However, when total chewing time is expressed per unit of ADF intake corrected for MBW, it remains unaffected by the level of dietary ADF intake. This suggests that the efficiency of chewing per unit of fiber intake may remain constant. Conversely, chewing efficiency declined with an increase in dietary ADF intake [92]. Specifically, chewing efficiency, measured as min/(g of ADFI × kg MBW), rose alongside ADF intake. This indicates that goats exhibited greater chewing efficiency as ADF intake increased, chewing only 71% of the ADF consumed compared to those on a low fiber diet. The relationship between chewing time and dietary fiber can be represented by the equation: total chewing time (min/day) = 33.11 + 30.13 ADF intake (%). For growing goats aged between 4 and 8 months, a dietary ADF level of 23% is recommended [9].

A diet rich in fiber supplies the necessary substrates that promote the proliferation of cellulolytic microbes and enhance salivation during eating and ruminating. The buffering capacity of salivation further elevates ruminal pH, which supports the growth of cellulolytic microbes and leads to the production of greater amounts of acetic and butyric acids. In primiparous lactating dairy goats, feeding forage with longer particle lengths resulted in a slight increase in ruminal pH and an improved acetate to propionate ratio [95]. As the intake of dietary fiber rose, the concentration of ruminal acetate also increased. Additionally, the acetate to propionate ratio improved with higher dietary ADF levels. Ruminal pH and the acetate to propionate ratio were linked to overall chewing activity. Furthermore, there was a clear relationship between ruminal pH and ruminal acetate concentrations [96].

Goats given a diet consisting of 15% corn bran took a longer time to ruminate compared to those that received 30% corn bran [97]. Additionally, providing animals with a diet with a higher proportion of forage relative to concentrate enhances both the chewing rates and the duration of eating and ruminating [98]. Conversely, a lower pH tends to promote the growth of amylolytic microorganisms, which produce a greater amount of propionic acid. The kinetics of cellulose digestion follow first-order kinetics, and the process is constrained by the availability of substrate rather than the cellulolytic capabilities of the resident microflora [99].

#### 4.8.2. Effect of Forage to Concentrate Ratio

The ratio of forage to concentrate (F:C) in the diets of small ruminants influences both feed intake and digestion. A reduction in the F:C ratio leads to a decrease in total chewing time, measured per unit of Neutral Detergent Fiber (NDF) and adjusted for MBW [7]. DM and OM digestibility showed a linear decrease as the F:C ratio increased in goats [100]. The CH_4_ emissions, measured in grams per day, grams per kilogram of body weight to the power of 0.75, as a percentage of gross energy intake, grams per kilogram of DMI, and grams per kilogram of OMI, also decreased linearly with an increase in the F:C ratio. However, the CH_4_ emissions expressed as grams per kilogram of digested DMI and OMI were not influenced by the F:C ratio [100].

High-concentrate diets led to improved nutrient digestibility, with the exception of ADF [101]. However, CP digestibility was only enhanced in the high-concentrate diets. In animals fed grass hay, nitrogen retention, ruminal NH3-N levels, and urinary purine derivative excretion increased as the concentrate in their diets rose, but this was not the case for those on alfalfa hay. The F:C ratio influenced ruminal pH but did not affect total VFA concentration. Ammonia-N levels remained consistent over time, while pH, VFA concentration, and protozoa counts varied. The impact of changing the F:C ratio from 70:30 to 30:70 in goat diets is contingent upon the type of forage used (grass hay versus alfalfa hay). Ramanzin [102] examined the influence of F:C on comparative digestion in sheep, goats, and fallow deer, utilizing three different F:C ratios of 10:90, 50:50, and 90:10, along with two feeding levels (45 and 90 g/kg M0–75 per day). Sheep exhibited the highest intakes and apparent digestibilities of the forage-rich diets, along with the longest mean retention times in the rumen. Goats, on the other hand, tended to select specific diet components, even with limited dietary allowances, resulting in lower food intakes compared to sheep [102]. Their apparent digestibility of the forage-rich diet was also lower, and their rumen mean retention times were shorter and less affected by dietary treatments [102].

Adding straw to high-concentrate total mixed rations for fattening goats can provide the necessary fiber to prevent ruminal acidosis and promote growth. Up to 20% of rice straw can be incorporated into goat diets, along with a concentrate supplement at 1% of body weight, while using tree foliage as the primary diet [103]. Simply offering fodder tree leaves is insufficient for achieving higher ADG in stall-feeding systems. Allowing small ruminants to ‘stall-graze’ on long barley straw will improve intake through selective feeding [104]. Allowing goats to reject 50% of the straw offered, instead of the conventional ad libitum refusal rate of 10 to 20%, resulted in increased dry-matter consumption of straw and a higher estimated digestible straw intake [104].

Treatment of straw and other novel feeds, along with the addition of a concentrate source, would enhance their utilization by small ruminants. Goats that were provided with treated straw at a 50% rate, combined with ammonia, consumed as much digestible straw as they did when fed the original straw in ample amounts. A botanical separation of the offered straw and the discarded straw revealed that goats have a preference for leaves over stems when the quantity available allows for selective feeding. Khurshid et al. [105] examined the impact of pelleted feed on a conventional total mix ration (TMR) that included 15% and 25% wheat straw on goat performance. Both pelleted TMR treatments resulted in increased feed intake and growth performance. The feed-to-gain ratio was superior in goats fed 25% straw compared to those receiving 15% straw. Feeding pelleted TMR with straw led to improved DMI and growth performance when compared to those on conventional TMR. The group receiving 15% straw outperformed the 25% straw group without causing any negative effects on blood metabolites, liver enzymes, or hematological parameters. Kids receiving concentrate pellets showed greater ADG, slaughter weight, dressing percentages, and enhanced carcass characteristics compared to the control group [105]. Supplementing growing kids with concentrate pellets at 1.5% of their live weight (LW) on the farm led to improved growth performance, better carcass traits, and resulted in leaner carcasses [106]. The intake of OM and CP rose with higher levels of concentrate supplementation in goats that were fed a mixture of *Prosopis cineraria* and *Albizia lebbeck* leaves in a 50:50 ratio [107]. In general, ADG increased with higher concentrate supplementation levels. Additionally, daily ME intake and the apparent digestibility of dry matter (DM), OM, CP, NDF, ADF, and cellulose were greater in supplemented lambs. All animals maintained a positive nitrogen (N) balance, which improved with increased concentrate supplementation [107].

### 4.9. Effects of Low-Quality Feeds on Growth and Carcass Composition

Feeding high-fiber NFs diets often leads to slower growth rates compared to high concentrate diets [56]. Goats given 12.3, 18.3, and 24.7% ADF (23.6, 33.0, and 43.4% NDF) exhibited growth rates of 45 and 45, 83, and 51, and 98 and 55 g/day, respectively. An increase in dietary fiber levels resulted in a reduction in fat content in both carcass and non-carcass portions of goats [56,108] suggesting the necessity to supplement their diet with some concentrate feeds.

Additionally, other factors such as genetics and climate must also be taken into account. Both supplementation and crossbreeding significantly improved carcass weight and primal cuts, while supplementation alone enhanced the dressing percentage, and crossbreeding led to an increase in shear force [109]. Regardless of breed, the inclusion of agricultural by-products resulted in better lipid oxidation stability and texture in fresh lamb, while lambs raised exclusively on pasture displayed healthier fatty acid profiles compared to those that were supplemented. These results indicate that both terminal sire crossbreeding and by-product supplementation can affect carcass characteristics and meat composition in lambs. Sheep and goats raised on pasture yielded lighter carcasses with reduced fat content [110]. In general, meat-producing sheep and goats on a high-energy diet exhibited enhanced juiciness, tenderness, and texture in their meat. However, consumer acceptance was lower due to the increased fat content in comparison to those finished on forage diets.

Various novel supplements may be used to improve small ruminants’ growth performance and meat quality. In lamb fed Mediterranean lupins diets, carcass and meat quality was comparable between lambs fed soybean meal or lupins, indicating that the lupins could be a potential alternative protein source [111]. Feeding *Nicotiana tabacum* cv. *Solaris* seed cake lowered lambs average daily gain and cold carcass yield but had no effect on feed intake. Seed cake had no negative effect on physico-chemical and nutritional characteristics of meat [112]. Pomegranate seed cake (PSC) PSC partially replaced cereals in growing lamb diets with no effects on their performance and carcass quantitative characteristics. PSC supplementation could improve the nutritional and functional properties of meat and subcutaneous fat, as indicated by the increase in essential fatty acids. Low inclusion levels of PSC could have a positive effect on antioxidant potential, and on nutritional and functional quality of meat, whereas high levels could possess an opposite effect [113].

### 4.10. Strategies to Enhance Utilization of Low-Quality Feeds

The limitations in the nutritional quality of feed resources negatively affect the production of small ruminants, an issue that must be addressed. A common strategy is to implement supplementation technology to mitigate both nutrient deficiencies and the effects of toxicity. Options for supplementation include high-quality protein and energy sources such as leguminous fodders, along with energy alternatives like molasses, cereal grains, by-products, and oilseeds. Additionally, protein meals, minerals, vitamins, and feed additives may be part of the mix. It is important that supplementation is customized to the specific protein content and mineral profiles of forages in each region [110]. Providing minerals and protein to sheep and goats consuming high-fiber, low-protein forages generally improve their intake and overall performance. Multi-nutrient blocks usually composed of molasses and urea can enhance rumen fermentation and supply the vital nutrients necessary to compensate for deficiencies in animals fed low-quality forages.

To enhance the utilization of low-quality feeds in small ruminants, several strategies can be implemented. One approach is grain supplementation, which decreases the duration animals spend on chewing and ruminating [95]. Providing concentrate supplementation to grazing lambs at four weeks of age for eight weeks led to improvements in BW, ADG, and a reduction in the costs associated with lamb production [114]. In goats given low-quality hay supplemented with molasses blocks containing urea, negative nitrogen balance was noted in goats that consumed blocks lacking urea [95]. The intake of nitrogen increased in goats that receive molasses blocks with urea, although urea supplementation did not influence DM or NDF intake or digestibility. Through regression analysis of CP intake and nitrogen balance derived solely from urea, a minimum CP requirement of 8% for maintenance in goats consuming low-quality forage with a urea-based supplement was established. Increasing the urea concentration in molasses blocks to 6% resulted in enhanced nitrogen intake, retention, and balance in goats [95].

### 4.11. Challenges of Feeding Pregnant Sheep and Goats on Low-Quality Feeds

Providing low-quality feed to pregnant small ruminants can have detrimental effects on both the dam and her offspring, necessitating supplementation for animals with inadequate nutrition. A higher nutritional intake during the final stages of pregnancy in sheep and goats led to improvements in the dam’s body weight, the rectal temperature of kids, the birth weight of lambs, and the weaning weight of both kids and lambs [115]. Furthermore, dams that received a high level of concentrates dedicated more time to grooming their young and were more supportive of their offspring’s suckling behavior. Lambs born to ewes with a high nutritional intake exhibited elevated serum total protein and globulin levels compared to those born to ewes on a standard diet. Increasing the concentrate mixture during late pregnancy enhances birth weight, boosts immunity, and decreases the likelihood of hypothermia [115].

Prenatal maternal nutrition significantly impacts fetal development. This potentially leading to enduring epigenetic modifications with protein intake during pregnancy affects the epigenetic regulation of fetal skeletal muscle via miRNA expression [116]. A moderate nutritional deficiency, approximately one-third, during mid-gestation resulted in stunted growth and development of the offspring, consequently diminishing carcass yield [117]. Nevertheless, the quality and composition of the meat, including amino acid and fatty acid profiles, were minimally impacted. Maternal undernutrition led to a decrease in body weight and size, ADG, and hot carcass weight of the kids, while tyrosine concentration increased. However, the proximate composition along with amino acid and fatty acid profiles remained unchanged in the restricted kids. In summary, maternal undernutrition during mid-gestation reduced the yield of kid meat without altering its quality and composition. Adipogenesis and myogenesis, which commence in early prenatal stages, appear to be highly responsive to nutritional influences, prompting numerous studies on fetal programming (the impact of the in-utero environment, such as the diet of the pregnant dam on the offspring) in cattle [118].

A significant increase in LWG, milk production, and lactation duration, along with a reduction in the length of post-partum anestrus in dams, was observed with high protein intake [119]. Additionally, a high protein diet enhanced the survival rates of kids, their weaning weight, final weight, LWG, dressing percentage, average body length, wither height, average heart girth, hot carcass weight, and ether extract content in the meat. The expression levels of the H-FABP gene were found to be higher in kids from those that were fed a high-protein diet compared to those from medium- and low-protein groups.

## 5. Conclusions

This review examines the dietary requirements of sheep and goats concerning various production factors. When utilizing established nutritional standards for sheep and goats that are typically fed novel and lower quality feeds, it is important to consider additional aspects such as energy expenditure, diet selection, grazing or browsing behavior, and environmental factors, especially ambient temperatures. Use of NFs could improve performance and meat production quality. For small ruminants in developing areas, implementing supplementation technology to address both nutrient deficiencies and toxic effects seems to be crucial, feasible, and effective.

## Figures and Tables

**Table 1:** Nutrient requirements of sheep and goats at various life stages [28].

Attributes	Sheep		Goats	
	CP%	TDN%	CP%	TDN%
Maintenance (mature female)	9.6	57.6	10	55
Late Gestation	11.2	66.7	11 *	60 *
Lactation	14.8 #	64.5 #	14 **	65 **
Early Weaned	14.5	75.8	14	68
Finishing (4–7 months)	11.7	77.1	-	-
Yearlings	9.1	57.6	12	65

# Nursing twins; * Average milk; ** High milk NRC [28,61].

**Table 2:** Comparison of some different estimations of energy and protein requirements of growing sheep [60].

BW, kg	ADG g/Day	Energy, MJ/Day			CP, g/Day		
		Paul et al. [60]	NRC [61]	Kearl [65]	Paul et al. [60]	NRC [61]	Kearl [65]
5	100	3.25	2.36	2.12	69	NS	45
10	200	5.90	6.05	4.54	132	123	90
15	200	7.02	8.14	6.05	145	134	121
20	200	8.71	10.12	7.56	NS	145	150
25	200	9.64	11.94	8.02	164	152	160
30	200	10.50	13.69	10.13	175	154	204

NS = Not stated.

## Data Availability

Not applicable.
